# Supplementary material for: Dietary Patterns during Pregnancy Are Associated with the Risk of Gestational Diabetes Mellitus: Evidence from a Chinese Prospective Birth Cohort Study
Source: Nutrients. 2019 Feb 15;11(2):405. doi: 10.3390/nu11020405 (PMC6412704; doi:10.3390/nu11020405)
Supplement: Supplementary file 1 [file nutrients-11-00405-s001.pdf]

Table S1. Complete list of the 21 food groups.

Food group	Food items
Rice	Rice, rice flour
Wheat flour and products	Noodle, bread, bun, flour, steamed twisted roll
Whole grains	Brown rice, millet, sorghum, black rice
Tubers	Potatoes, sweet potatoes,
Red meat	Pork, pork ribs, pork fat, beef, lamb, donkey, rabbit
Poultry	Chicken, duck, goose
Organ meats	Liver, kidney, blood, large intestine
Freshwater fish	Carp, squid, grass carp, chub, catfish
Marine fish	Hair tail, yellow croaker, squid, herring, striped bass
Shrimps, crabs and mussels	Shrimp, crab, scallop, oyster
Seaweed	Fresh or dried seaweed
Milk	Cow milk, goat milk
Dairy products	Yogurt, cheese
Eggs	Egg, duck egg, goose egg
Beans and bean products	Broad bean, soy, tofu, dried tofu, soy milk
Fruits	Apple, pear, orange, banana, grape, peach, watermelon, cherry, jujube
Vegetables	Spinach, cabbage, Chinese cabbage, celery, rape, oil wheat, western lettuce, chives, tomato, cucumber, carrot, winter melon, onion, green paper, broccoli, mushroom
Nuts	Peanut, walnut, sesame, cashew, almond, sunflower seed, pistachio
Pastry and candy	Jelly, jam, chocolate, honey, sugar, candy, mooncake
Fried foods	Chips, French fries, fritters
Sweat Beverages	Carbonated drinks, sweet tea, soft drinks, fruit juice

Table S2. Associations of intake of foods and food groups (expressed as z-scores) from food frequency questionnaires (FFQ) with risk for GDM and difference in mean plasma glucose level during oral glucose tolerance testing (OGTT)

	GDM, OR (95% CI)	Plasma glucose level, β (95% CI)		
Foods from FFQ	Reference= Non-GDM	Fasting	1 hour after OGTT	2 hours after OGTT
Rice	0.76 (0.65, 0.89) **	-0.03 (-0.07, 0.00) *	-0.13 (-0.23, -0.02) *	-0.07 (-0.15, 0.01)
Wheat flour and products	1.02 (0.88, 1.19)	0.01 (-0.03, 0.04)	0.01 (-0.09, 0.11)	0.03 (-0.05, 0.11)
Whole grain	1.02 (0.88, 1.18)	-0.01 (-0.04, 0.03)	0.11 (0.01, 0.22) *	0.12 (0.04, 0.20) **
Tubers	0.88 (0.74, 1.03)	-0.01 (-0.05, 0.02)	-0.05 (-0.16, 0.05)	-0.01 (-0.09, 0.07)
Red meat	0.94 (0.80, 1.10)	0.01 (-0.02, 0.04)	-0.02 (-0.12, 0.08)	-0.02 (-0.10, 0.06)
Poultry	0.92 (0.78, 1.09)	-0.01 (-0.04, 0.02)	-0.05 (-0.15, 0.05)	-0.04 (-0.12, 0.04)
Organ meats	0.95 (0.81, 1.12)	-0.02 (-0.05, 0.01)	0.05 (-0.05, 0.16)	0.01 (-0.07, 0.09)
Freshwater fish	1.04 (0.89, 1.20)	0.01 (-0.03, 0.04)	0.08 (-0.02, 0.19)	0.03 (-0.05, 0.11)
Marine fish	1.05 (0.91, 1.21)	0.00 (-0.04, 0.03)	0.05 (-0.05, 0.16)	0.06 (-0.02, 0.14)
Shrimps, crabs and mussels	1.10 (0.95, 1.26)	0.00 (-0.04, 0.03)	0.09 (-0.02, 0.19)	0.05 (-0.03, 0.13)
Seaweed	1.03 (0.89, 1.19)	-0.01 (-0.04, 0.02)	0.00 (-0.10, 0.10)	0.02 (-0.05, 0.10)
Milk	1.05 (0.91, 1.22)	0.02 (-0.02, 0.05)	0.03 (-0.07, 0.14)	-0.01 (-0.09, 0.07)
Dairy products	1.10 (0.95, 1.28)	0.04 (0.00, 0.07) *	0.05 (-0.04, 0.16)	0.09 (0.01, 0.17) *
Eggs	0.96 (0.82, 1.13)	-0.01 (-0.04, 0.03)	0.04 (-0.06, 0.15)	0.05 (-0.03, 0.13)
Beans and products	0.86 (0.72, 1.02)	0.00 (-0.03, 0.03)	0.00 (-0.10, 0.11)	0.00 (-0.09, 0.08)
Fruits	0.77 (0.65, 0.90) **	-0.06 (-0.09, -0.02) **	-0.10 (-0.21, 0.01)	-0.12 (-0.21, -0.04) **
Vegetables	0.85 (0.73, 1.00) *	-0.04 (-0.07, -0.01) *	-0.07 (-0.18, 0.03)	-0.07 (-0.15, 0.01)
Nuts	1.05 (0.90, 1.21)	0.01 (-0.02, 0.04)	0.03 (-0.07, 0.14)	0.03 (-0.05, 0.11)
Pastry and candy	0.96 (0.82, 1.12)	0.02 (-0.02, 0.05)	-0.01 (-0.12, 0.09)	-0.04 (-0.12, 0.04)
Fried foods	0.82 (0.65, 1.04)	0.02 (-0.01, 0.05)	-0.02 (-0.13, 0.08)	-0.06 (-0.14, 0.02)
Sweat Beverages	1.00 (0.87, 1.16)	0.03 (-0.01, 0.06)	-0.04 (-0.14,0.07)	0.00 (-0.08, 0.08)

Adjusted for all other food items, pre-pregnancy BMI, pre-pregnancy BMI square, age, parity, family income, education level, ethnicity, smoking status, total energy intake, physical activity. OGTT, oral glucose tolerance test. GDM, gestational diabetes mellitus. ***: p<0.001; **p<0.01; *p<0.05.
